# X-linked severe combined immunodeficiency complicated by disseminated bacillus Calmette-Guérin disease caused by a novel pathogenic mutation in exon 3 of the IL2RG gene: a case report and literature review

**DOI:** 10.3389/fimmu.2024.1453046

**Published:** 2024-08-08

**Authors:** Chunxue Jiang, Yunhan He, Xin Chen, Fei Xia, Feng Shi, Xuewen Xu, Tingting Sun, Kai You

**Affiliations:** ^1^ Department of Pediatrics, Shengjing Hospital of China Medical University, Shenyang, China; ^2^ Computer Center, Shengjing Hospital of China Medical University, Shenyang, China; ^3^ Department of Urology, Shengjing Hospital of China Medical University, Shenyang, China

**Keywords:** X-linked severe combined immunodeficiency, interleukin-2 receptor gamma-chain gene, disseminated bacillus Calmette-Guérin disease, case report, systemic review

## Abstract

X-linked severe combined immunodeficiency (X-SCID), caused by mutations in the gamma-chain gene of the interleukin-2 receptor (IL2RG), is a prevalent form of SCID characterized by recurrent and fatal opportunistic infections that occur early in life. The incidence of disseminated bacillus Calmette-Guérin (BCG) disease among children with SCID is much higher than in the general population. Here, we report the case of a 4-month-old male infant who presented with subcutaneous induration, fever, an unhealed BCG vaccination site, and hepatosplenomegaly. Metagenomic next-generation sequencing in blood, and the detection of gastric juice and skin nodule pus all confirmed the infection of *Mycobacterium tuberculosis*. Lymphocyte subset analysis confirmed the presence of T-B+NK immunodeficiency. Whole-exome and Sanger sequencing revealed a novel microdeletion insertion mutation (c.316_318delinsGTGAT p.Leu106ValfsTer42) in the IL2RG gene, resulting in a rare shift in the amino acid sequence of the coding protein. Consequently, the child was diagnosed with X-SCID caused by a novel mutation in IL2RG, complicated by systemic disseminated BCG disease. Despite receiving systemic anti-infection treatment and four days of hospitalization, the patient died three days after discharge. To the best of our knowledge, this specific IL2RG mutation has not been previously reported. In our systemic review, we outline the efficacy of systemic anti-tuberculosis therapy, hematopoietic stem cell transplantation, and gene therapy in children with SCID and BCG diseases caused by IL2RG gene mutation.

## 1 Introduction

Severe combined immunodeficiency disease (SCID), a rare and fatal primary immunodeficiency disease caused by genetic defects, is characterized by a severe deficiency in T cells, B cells, and NK cells, or functional abnormalities, resulting in combined defects in humoral and cellular immunity. The incidence rate of SCID is approximately 1.5 per 100,000 live births (1, 2). Patients with SCID often develop severe opportunistic infections within 6 months of life, and without hematopoietic stem cell transplantation treatment, most die within their first year. X-linked severe combined immunodeficiency (X-SCID), caused by mutations in the interleukin-2 receptor gamma chain (IL2RG) gene, is a common type of SCID, accounting for approximately 50% to 60% of cases (3).

Disseminated bacillus Calmette-Guérin (BCG) disease is one of the most severe adverse reactions to BCG vaccination though relatively rare. The overall incidence rate of BCG disease is estimated to be 1–3.4 per 100 million persons, primarily affecting children with immunodeficiency disease and associated with high mortality (4, 5). This study reports a case of X-SCID complicated by systemic disseminated BCG disease caused by a novel mutation in IL2RG and reviews the relevant literature to summarize the clinical and genotypic characteristics of this condition. The main clinical information and genetic loci of this case are highlighted to improve the understanding of early identification and disease progression, facilitate timely initiation of immune reconstitution therapy, and assist genetic counseling efforts.

## 2 Case presentation

A four-month-old male was admitted to our hospital with subcutaneous induration persisting for 20 days, fever for 10 days, and poor response for 1 day. The patient was the second child of healthy, non-consanguineous parents, born full-term via cesarean section, with a birth weight of 3600 g. Two days after birth, the patient received the BCG vaccine (Chengdu Biotech, batch number: 202207a034), administered to the lower edge of the deltoid muscle in the left upper arm. At 1 week old, a hemangioma measuring approximately 5 cm was noted behind the right ear. At 3 months old, subcutaneous nodules appeared on the abdomen and later spread across the body. Subsequently, the patient developed fever, mental fatigue, poor feeding, and repeated bleeding and scabbing at the BCG vaccination site. Despite these issues, the patient’s growth and development were within the normal ranges, and the patient’s 6-year-old sister was healthy.

**Figure 1:**
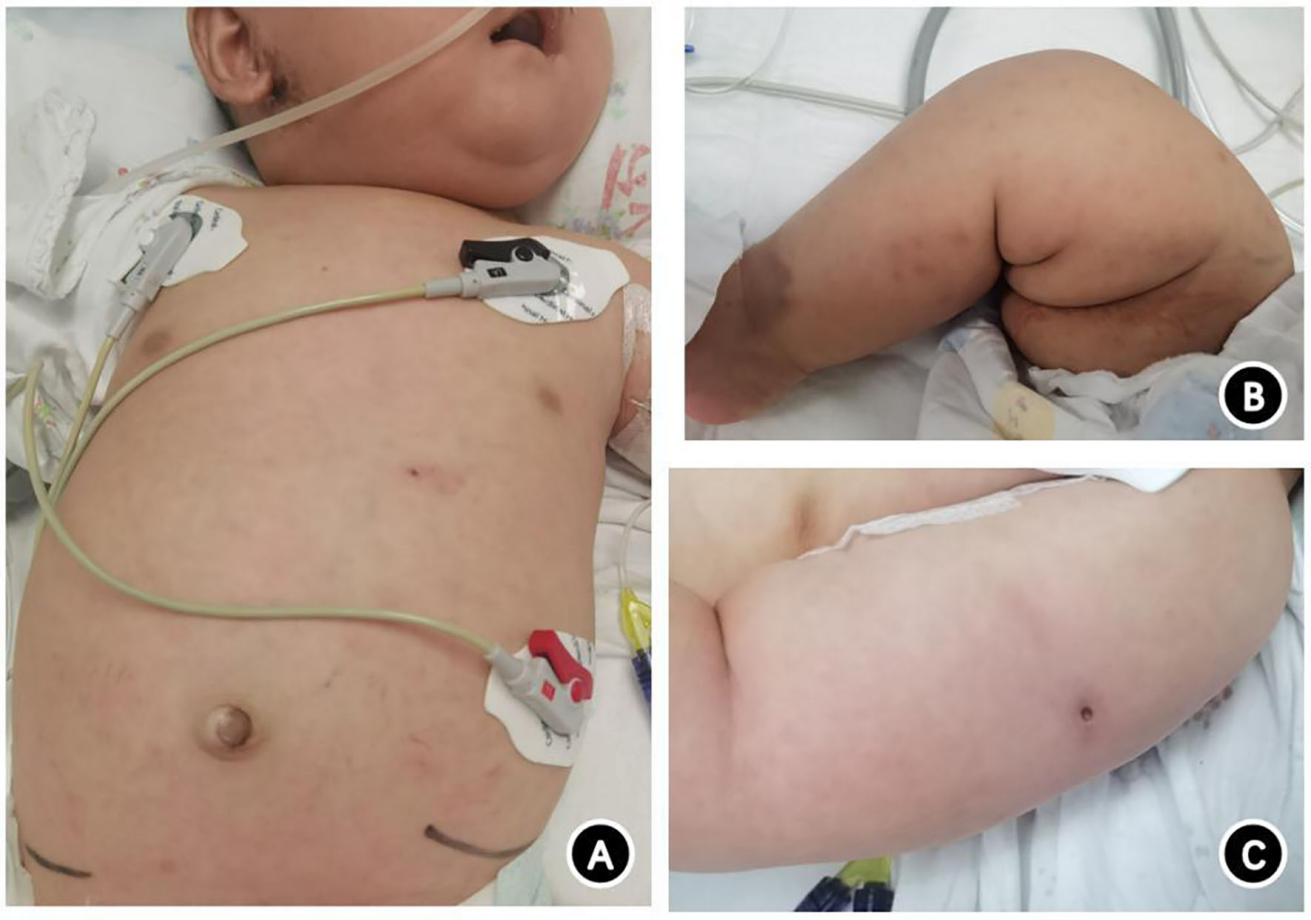
Photograph of a 4-month-old patient with SCID complicated by systemic disseminated BCG disease caused by a novel IL2RG gene mutation. **(A)** Scattered subcutaneous nodules on the trunk, with ulceration and scabbing visible below the ear and in the upper left abdomen. **(B)** Scattered rashes and purplish-blue subcutaneous nodules on the lower limbs, with some scabs forming. **(C)** Unhealed BCG vaccination site with liquid leakage.

Upon hospital admission, the patient weighed 7.9 kg (P50–P75), measured 146 cm (P25–P50) in length, and exhibited a poor mental state. Scattered blue-purple subcutaneous nodules (<1 cm × 1 cm in size) were visible throughout the body. These did not fade under pressure, and some were ulcerated and scabbed (**Figures 1A, B**). The BCG vaccination site exhibited rupture with fluid exudation (**Figure 1C**). No superficial lymph node enlargement was observed, and scattered moist rales were present in both lungs. The abdomen was distended, with the liver lower edge palpable 6 cm below the right costal margin and the spleen lower edge palpable 4 cm below the left costal margin.

Initial laboratory test results were as follows: white blood cell count, 2.54×109/L (4.3×109–11.3×109); lymphocyte count, 0.1×109/L (1.5×109–4.6×109); hemoglobin, 71 g/L (118–156); platelet count, 64×109/L (167×109–453×109); C-reactive protein, 154 mg/L (0–5); serum ferritin, 3737 ng/ml (13–84); D-dimer, 14234 μg/L (0–500); interleukin (IL)-6, >5000 pg/ml (0.373–0.463); procalcitonin, 6.8 ng/ml (<0.05); serum aspartate aminotransferase, 149 U/L (<30); and alanine aminotransferase, 45 U/L (<30). Immunological assessment showed an immunoglobulin G level of 0.84 g/L (7.51–15.6), immunoglobulin A level of <0.0667 g/L (0.82–4.53), and immunoglobulin M level of 0.102 g/L (0.46–3.04). Lymphocyte subset analysis revealed that the percentage of lymphocytes was 6.0% (16.8–43.4), with T, natural killer, and B lymphocytes accounting for 0.54% (55–84), 1.35% (7–36), and 93.26% (5–10) of lymphocytes, respectively. Helper and suppressor T lymphocytes accounted for 0.27% (13–41) and 0.27% (13–27) of total lymphocytes, respectively (**Table 1**). Acid-fast-positive bacteria were detected in the skin nodule pus puncture smear, and the proB gene test for *M. tuberculosis* in gastric juice was positive. The T cell spot test (T-SPOT) for tuberculosis (TB) infection and bacterial culture smear of cerebrospinal fluid were negative.

**Table 1:** Immunological data upon admission and before discharge.

Variable	Upon admission	Before discharge	Reference range
Serum immunoglobulins			
Immunoglobulin G (g/L)	0.84	-	7.51-15.6
Immunoglobulin M (g/L)	0.102	-	0.46-3.04
Immunoglobulin A (g/L)	<0.0667	-	0.82-4.53
Lymphocyte subsets			
CD3+ (%)	0.54	2.44	55–84
CD3+ (cells/μL)	1	4	690–2540
CD3+CD8+ (%)	0.27	1.33	13–41
CD3+CD8+ (cells/μL)	0	2	190–1140
CD3+CD4+ (%)	0.27	0.37	31–60
CD3+CD4+ (cells/μL)	0	1	410–1590
CD16+CD56+ (%)	1.35	0.05	7–36
CD16+CD56+ (cells/μL)	2	0	90–590
CD19+ (%)	93.26	97.13	5–10
CD19+ (cells/μL)	181	145	90–660
CD4+/CD8+	1.00	0.28	0.71–2.78

**Figure 2:**
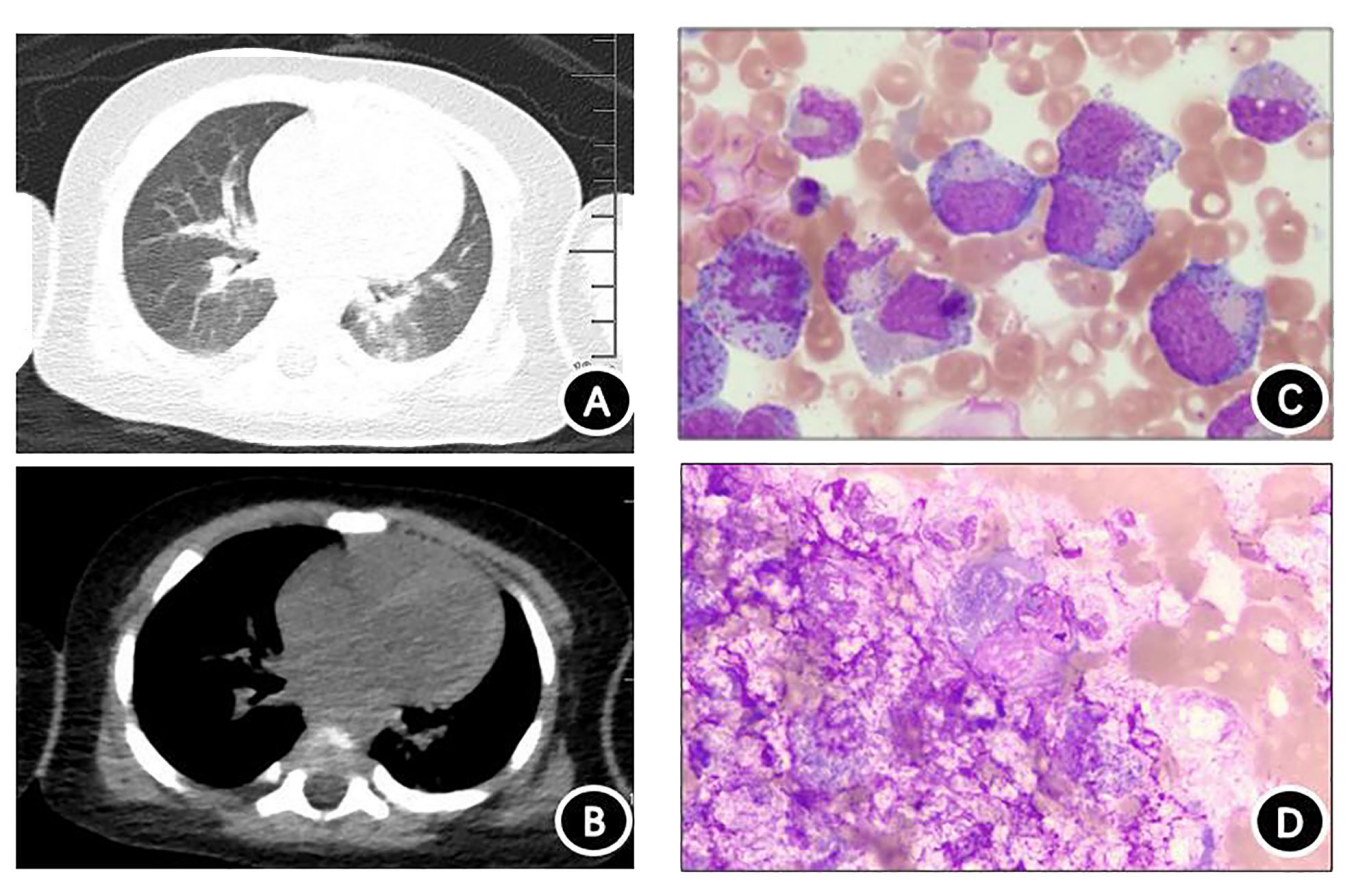
Chest computed tomography (CT) and cytological examination. **(A)** Chest CT lung window. **(B)** Chest CT mediastinal window showing scattered inflammation in both lungs. **(C)** Bone marrow specimen demonstrating significant proliferation and activation of bone marrow, with toxic particles and vacuolar degeneration visible in granulocytes. Red blood cells are mainly composed of middle- and late-stage erythrocytes, and no plate-producing megakaryocytes are present. **(D)** Rash imprint showing approximately 18% of suspected Langerhans tissue cells, with abundant cytoplasm filled with small grayish-purple particles. The nucleoli are circular or irregular in shape, and the cytoplasm is thick and loose.

Chest computed tomography (CT) revealed slight inflammation in both lungs (**Figures 2A, B**). Abdominal CT indicated liver and spleen enlargement with some fluid accumulation in the abdominal and pelvic cavities. Bone marrow examination revealed active hyperplasia of bone marrow, neutrophils with toxic granulation and vacuoles, iron-deficient erythroid cells, and poor platelet production in megakaryocytes (**Figure 2C**). Langerhans cells were found on the skin print (**Figure 2D**) and the bovine *M. tuberculosis* complex was detected in the blood using metagenomic next-generation sequencing (mNGS) technology.

After admission, the patient was empirically treated with broad-spectrum antibiotics (Shupushen combined with linezolid) and received immunoglobulin infusion and red blood cell suspension as supportive therapy.

**Figure 3:**
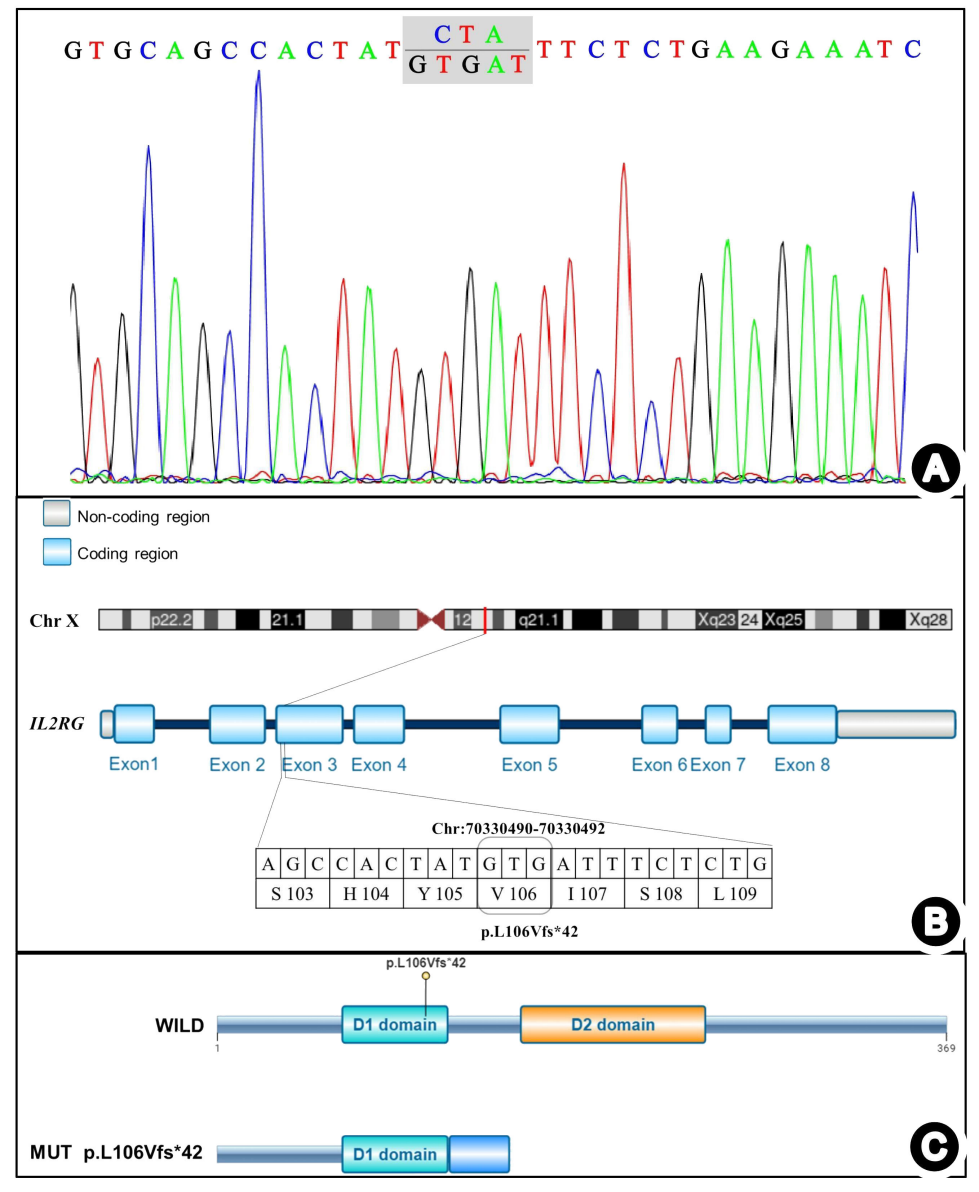
IL2RG gene mutation detected in the present case. **(A)** Sanger chromatogram. **(B)** Schematic diagram of the mutation sites. **(C)** Schematic diagram of the mutation protein.

Lymphocyte subset analysis revealed low T and NK lymphocyte counts, alongside increased B lymphocytes, confirming T-B+NK- immunodeficiency. Therefore, genetic testing was performed on the patient, his parents, and his older sister to identify the molecular defects and genetic risk of immunodeficiency. Whole-exome and Sanger sequencing revealed a microdeletion insertion mutation in exon 3 of the IL2RG gene (chrX: 70330490–70330492), resulting in a frameshift mutation at the amino acid level of the coding protein IL2RG (NM_000206.3) gene, c.316_318 delins GTGAT p.Leu106ValfsTer42 (**Figures 3A, B**). This mutation was expected to cause the 106th amino acid in the protein amino acid sequence to change to valine from leucine and result in an early stop codon at the 147th amino acid, terminating translation at the 42nd amino acid and producing a truncated protein that differs from the original sequence (**Figure 3C**). Currently, there are no reports in the literature on this rare frameshift mutation, either domestically or internationally. Experimental data suggested that the mutation was inherited from the mother of the patients (heterozygous state), while the father did not carry this mutation. NGS of the patient’s sister identified no mutation, indicating she was wild-type at the c.316_318 locus of the IL2RG gene.

The patient was ultimately diagnosed with SCID complicated by systemically disseminated BCG disease. While the attending physician recommended the patient receive systemic anti-TB treatment and immune reconstitution at a specialized hospital, the parents refused the proposal and the child was discharged on the fourth day after admission. Unfortunately, the patient died three days later.

## 3 Systematic review

A comprehensive search was conducted in PubMed, Web of Science, ClinVar, Embase, and Medline databases using the search terms “IL2RG,” “BCG disease,” and “SCID.” This search yielded 11 reports and 24 children of X-SCID complicated with BCG disease caused by IL2RG gene mutation, published prior to June 2024 (**Table 2**). Among the 25 children (current and previous studies), all were male, with ages ranging between 1–10 months and a median age of 4.56 months at diagnosis. Most patients (n=20, 80%) presented with skin lesion symptoms, such as unhealed BCG vaccination sites, scattered rashes, subcutaneous nodules, skin rash, and eczema. Additionally, patients had pneumonia (n=22), persistent hepatosplenomegaly (n=17), persistent diarrhea (n=11), sepsis (n=8), and failure to thrive (n=6) symptoms. Lymph node enlargement was observed in only one child prior to transplantation. The most commonly affected organs were the lungs, followed by the liver and the spleen.

**Table 2:** Children with SCID caused by IL2RG gene mutation complicated by BCG disease.

No.	Sex	Age (months)	Clinical features	Mutation point of IL2RG gene	Gene mutation type	Nucleotide change	Treatments	Outcomes	Ref.
P1	Male	4m	Unhealed BCG vaccination site, subcutaneous induration, fever, hepatosplenomegaly, pneumonia	Exon 3	Frameshift mutation	c.316_318delinsGTGAT p.Leu106ValfsTer42	Shupushen combined with linezolid, infusion of immunoglobulin, and suspension of red blood cells.	Deceased	Present case
P2	Male	4m	Unhealed BCG vaccination site, scattered pustules, rash scabbed, fever, hepatosplenomegaly, pneumonia, dyspnea, multiple-organ dysfunction, disseminated intravascular coagulation, secondary hemophagocytic syndrome, septic shock, hepatic VOD, GVHD, subcutaneous induration	Exon 2	Deletion mutation	c.139_148delACTGACTCCC	Anti-tuberculous treatment, infusion of bone marrow and peripheral blood stem cells, defibrotide, methylprednisolone, CSA, IVIG, imipenem cilastatin, cefoperazone sulbactam sodium, methylprednisolone, ibuprofen	Symptom improvement	(6)
P3	Male	5m	Unhealed BCG vaccination site, tender swelling in the right lower forearm, hepatosplenomegaly, pneumonia, skin-colored soft nodules, cough, reactive lymphohistiocytic proliferation, granuloma	Exon 6	Nonsense mutation	c.820_823dup p.Ser275Asnfs∗29	Intravenous immunoglobulin, sulfamethoxazole-trimethoprim, isoniazid, rifampin, vancomycin, piperacillin, tazobactam, azithromycin, amphotericin B liposomal, HSCT	Symptom improvement	(7)
P4	Male	4m	Hemophagocytic syndrome, fever, cough, papule, pneumonia, unhealed BCG vaccination site, dyspnea, hepatosplenomegaly, abdominal dropsy	Exon 6	Missense mutation	c.854G > A, p.Arg285Gln	CPAP, vancomycin, meropenem, IVIG, anti-tuberculous treatment, etoposide, dexamethasone	Deceased	(8)
P5	Male	8m	Fever, cough, pigmented papules, moderate oral thrush, unhealed BCG vaccination site, scattered pustules, pneumonia, massive hematemesis, melena, Pseudomonas aeruginosa sepsis	Exon 2	Missense mutation	c.136G>C	IVIG, fluid resuscitation, antibiotics	Deceased	(9)
P6	Male	5m	BCG site ulceration, pneumonia, persistent diarrhea, generalized papular rash	Exon 1	Frameshift mutation	c.8_9insA	NA	Deceased	(10)
P7	Male	6m	Recurrent pneumonia, otitis media, ulceration at BCG site, hepatosplenomegaly	Exon 8	Nonsense mutation	c.964C>T	NA	Deceased	(11)
P8	Male	4.5m	Oral thrush, recurrent pneumonia, diarrhea, failure to thrive, BCG site ulceration	Exon 1	Nonsense mutation	c.67delC	NA	Symptom improvement	(10)
P9	Male	10m	Recurrent diarrhea, pneumonia, otitis media, failure to thrive, BCG site ulceration, hepatosplenomegaly, generalized adenopathy, erythroderma, eosinophilia (Omenn syndrome)	Exon 2	Nonsense mutation	c.202G>T	NA	Deceased	(12)
P10	Male	6.5m	Recurrent pneumonia, BCG site ulceration, meningitis, hepatosplenomegaly, pancytopenia, transaminitis (HLH)	Exon 3	Missense mutation	c.515T>G	NA	Deceased	(10)
P11	Male	7m	Pneumonia, diarrhea, sepsis, multiple liver and spleen abscesses, oral candidiasis, hepatosplenomegaly, liver injury, anemia, skin rash	Exon 3	Disruption of RNA splicing	g.IVS3-15A>G	NA	Deceased	(13)
P12	Male	6m	Pneumonia, diarrhea, multiple liver and spleen abscesses, oral candidiasis, bone tuberculosis, hepatosplenomegaly, anemia, hypoalbuminemia, patent foramen ovale, eczema	Exon 3	Missense mutation	c.304T>C	NA	Deceased	(13)
P13	Male	4.56m	URI, pneumonia, diarrhea, hepatomegaly, failure to thrive/malnutrition, hypoalbuminemia, eczema	Exon 3	Nonsense mutation	c.312C>T	NA	Deceased	(13)
P14	Male	4.5m	Pneumonia, sepsis, urinary tract infection, oral candidiasis, oral ulcer, lymph node tuberculosis, hepatosplenomegaly, anemia, failure to thrive/malnutrition, thrombocytopenia, renal injury, myocardial injury, pleural effusion, skin rash	Exon 3	Missense mutation	c.385T>C	NA	Deceased	(13)
P15	Male	5m	Pneumonia, diarrhea, sepsis, purulent meningitis, liver and spleen tuberculosis, CMV infection, hepatosplenomegaly, anemia, hypoalbuminemia, hemophagocytic syndrome, bilateral subdural effusion, hydropericardium, patent foramen ovale	Exon 5	Missense mutation	c.690C>T	NA	Deceased	(13)
P16	Male	5m	Diarrhea, oral candidiasis, hepatosplenomegaly, anemia, thrombocytopenia, skin rash	Exon 5	Nonsense mutation	c.738G>A	NA	Deceased	(13)
P17	Male	4m	URI, pneumonia, diarrhea, sepsis, CMV infection, hepatosplenomegaly, anemia, thrombocytopenia, hypoalbuminemia, skin rash	Exon 6	Disruption of RNA splicing/Missense mutation	c.868G>A	NA	Deceased	(13)
P18	Male	4m	Pneumonia, diarrhea, multiple liver and spleen abscesses, hepatosplenomegaly, liver injury, thrombocytopenia, hypoalbuminemia, anemia, hydropericardium, patent foramen ovale, skin rash	Exon 7	Deletion, frameshift mutation	g.IVS7-72 to IVS8-11del487	NA	Deceased	(13)
P19	Male	4m	URI, pneumonia, diarrhea, sepsis, anemia, skin rash	NA	NA	NA	NA	Deceased	(13)
P20	Male	1m	Pneumonia	Exon 5	Missense mutation	c.679T>C	Anti-tuberculous treatment and ciprofloxacin	Symptom improvement	(14)
P21	Male	1m	Pneumonia, diarrhea, sepsis, developmental delay	Exon 2	Missense mutation	c.220T>G	Anti-tuberculous treatment and ciprofloxacin	Improved	(14)
P22	Male	3m	Fever, red papules, hepatosplenomegaly, sepsis, pneumonia, coagulopathy, mild disturbance of consciousness, meningeal irritation sign, HPS	Exon 3	Missense mutation	c.391C>T; p.Gln131Ter	Nasal high-flow ventilation, anticoagulant therapy, imipenem, dexamethasone, anti-tuberculous treatment, levofloxacin, UCBT, busulfan, cyclophosphamide, immunoglobulin, etanercept	Improved	(15)
P23	Male	4m	Fever, BCG site ulceration, axillary lymphadenopathy	Exon 8	Deletion mutation	c.903_910del;p.Glu302ArgfsX110	Anti-tuberculous treatment	Improved	(16)
P24	Male	5m	Fever, cough, respiratory distress, oral thrush, bilateral pulmonary rales, hepatosplenomegaly, conjunctivitis, otitis media, pneumonia	Exon 5	Missense mutation	p.Arg226His	Trimethoprim-sulfamethoxazole, anti-tuberculous treatment, antibacterial and antifungal prophylaxis, immunoglobulin replacement therapy, BMT, treosulfan, fludarabine, ampath, linezolid, steroids	Improved	(17)
P25	Male	5m	Decreased appetite, pneumonia, hypogammaglobulinemia, tachypnea	NA	NA	NA	UCBT, broad-spectrum antibiotics, anti-tuberculous treatment	Improved	(18)

X-SCID, X-linked severe combined immunodeficiency; IL2RG, interleukin-2 receptor; BCG, bacillus Calmette-Guérin; SCID, severe combined immunodeficiency disease; T-SPOT, T cell spot test; TB, tuberculosis; CT, chest computed tomography; mNGS, metagenomic next-generation sequencing; LCH, Langerhans cell histiocytosis.

NA, Not Applicable.

The 23 children from whom IL2RG gene mutation sites were reported had mutations exclusively located in the coding region and all were distinct from each other. These consisted of the following mutations: exon 1 (n=2), exon 2 (n=4), exon 3 (n=7), exon 5 (n=4), exon 6 (n=3), exon 7 (n=1), and exon 8 (n=2). Only 22 children were reported about gene mutation type, 16 were point mutations (10 missense, 4 nonsense, and 2 splicing mutations), 2 were frameshift mutations caused by deletion insertion, 2 were deletion mutations, 1 contained both splicing and missense mutations, and 1 contained both deletion and frameshift mutations.

Among the 25 children, 8 received hematopoietic stem cell transplantation and 1 received gene therapy. In addition, all 9 patients received systematic anti-tuberculosis treatment (isoniazid, rifampicin, ethambutol, etc.). At the last follow-up, all 9 patients who underwent these treatments were still alive. The remaining 16 children, who did not receive immune reconstitution therapy or gene therapy, died within 1 month of diagnosis.

## 4 Discussion

In this report, we present a case of a 4-month-old male infant diagnosed with systemic disseminated BCG disease and X-SCID caused by a novel and rare frameshift mutation in IL2RG, which was identified using whole-exome sequencing. Moreover, we reviewed and summarized the clinical characteristics and genetic findings from previously reported cases of children with SCID caused by mutations in the IL2RG gene complicated by BCG disease.

IL2RG, located on the human X chromosome, q13.1, comprises 8 exons and 4,145 base pairs and encodes the interleukin-2 receptor gamma chain. This protein is essential for various cytokine receptors including IL-2, IL-4, IL-7, IL-15, and IL-21 (19), which regulate the differentiation and development of T lymphocytes, NK lymphocytes, and other cells. Mutations in IL2RG disrupt cytokine signaling, leading to developmental arrest of T lymphocytes, which further causes B lymphocyte dysfunction, ultimately leading to humoral and cellular immune deficiencies, namely X-SCID. In the present case, the patient exhibited clinical symptoms at 3 months of age and died within 4 weeks of onset. Similarly, all the 16 children in the literature who did not undergo immune reconstitution treatment or gene therapy died within 1 month of disease onset.

The ClinVar database catalogs over 200 pathogenic mutations in IL2RG, including missense, nonsense, frameshift, and splice site mutations, predominantly located in exons 3, 4, and 5, and mainly comprising missense mutations. In our report, we observed a novel frameshift mutation that has not yet been previously reported. As mentioned earlier, most IL2RG pathogenic mutations lead to severe T-cell defects, further causing typical SCID characterized by T-B+NK-immunodeficiency. However, atypical X-SCID can manifest as T+B+NK- immunodeficiency with milder immune deficiency (20). In the present case, the patient’s mother was heterozygous with a normal phenotype, while the father and elder sister were wild-type, consistent with the inheritance pattern of an X-linked recessive genetic disorder. The patient exhibited T-B+NK immunodeficiency, and the onset of infection occurred between 3 and 6 months after birth, indicating that SCID caused by a rare frameshift mutation is a typical feature of X-SCID presentation. CD45Ra/Ro or detailed T-cell immune typing analysis was not conducted for the patient; therefore, we were unable to perform an in-depth assessment of the differentiation and function of immature and memory T-cell subsets, which is crucial for understanding immune dysfunction (21). According to the guidelines of the American College of Medical Genetics and Genomics, combined with the clinical phenotype and family analysis of the patient, this mutation meets the criteria for PVS1+PM2 pathogenicity classification and is considered a potential pathogenic mutation (22, 23).

SCID is prone to severe opportunistic infections caused by cytomegalovirus, Candida, Pseudomonas aeruginosa, Pneumocystis jirovecii, and BCG. BCG vaccination in children with SCID may lead to life-threatening systemic disseminated BCG disease. Studies suggest that 7% to 34% of children with SCID develop disseminated BCG disease, with the risk being 33,000 times higher than in those without SCID (5). Common manifestations of systemically disseminated BCG include rupture and pus at the vaccination site, formation of skin masses, persistent hepatosplenomegaly, enlarged lymph nodes, and musculoskeletal pain. Due to severe lymphocyte defects in children with SCID, lymph node enlargement may not be clinically evident in patients with BCG disease, with only one child presenting with lymph node enlargement among the 25 cases included in the literature review. In the present case, the patient experienced repeated bleeding and scabbing at the BCG vaccination site and subcutaneous nodules throughout the body. The diagnosis of systemic disseminated BCG disease was based on clinical manifestation, skin nodule pus puncture smear, proB gene test in gastric juice, and mNGS, which is highly sensitive for pathogen detection (24). In cases without specific clinical manifestations or where it is difficult to detect pathogens through these conventional methods, the T-SPOT test, an interferon-γ detection test, is a TB detection method recognized by the World Health Organization (25, 26). However, the T-SOPT test in the present case was negative, likely due to severe T lymphocyte deficiency impairing interferon-γ release. In addition, some children with SCID complicated by disseminated BCG disease may exhibit symptoms similar to Langerhans cell histiocytosis (LCH), such as body rashes, nodules, digestive issues, and bone involvement, potentially leading to misdiagnosis in the early disease stages (27). Although Langerhans cells were detected in the patient’s skin print, classic LCH signs such as bone destruction were absent, ruling out an LCH diagnosis.

Systemic anti-TB therapy is essential for treating systemic disseminated BCG disease, with simultaneous immune reconstitution in patients with concurrent SCID. Currently, hematopoietic stem cell transplantation is the primary method of immune reconstitution, followed by gene therapy (28). In the literature, all 9 patients who underwent immune reconstitution therapy or gene therapy survived, while the remaining 16 children who did not receive these treatment died. Additionally, multiple studies have shown that the survival rate of children with SCID who undergo hematopoietic stem cell transplantation within 3 months after birth exceeds 90% (1, 2, 29); thus, early identification and diagnosis of SCID are crucial for improving prognosis. However, owing to the lack of characteristic clinical manifestations in the early stages, unless there is a known family history, most children with SCID can only be diagnosed after 3 months of age, delaying timely immune reconstitution. Therefore, newborn screening during the neonatal period is crucial for detecting SCID early to facilitate prompt immune reconstitution and mitigate complications from the BCG vaccination. Notably, the T cell receptor excision circle test can specifically recognize naïve T cells, which aids in screening for SCID and T lymphocyte depletion caused by other reasons (30, 31). Alternatively, the kappa-deleting recombinant excision circle test, a screening method for identifying B cell defects, can also be used for newborn screening for primary immunodeficiency (32).

## 5 Conclusion

We report a case of SCID complicated by BCG disease caused by a novel IL2RG gene mutation and present a literature review that outlines the benefits of immune reconstitution and gene therapy in treating patients with SCID complicated by BCG disease.

## Author contributions

CJ: Conceptualization, Data curation, Formal analysis, Methodology, Project administration, Writing – original draft, Writing – review & editing. YH: Conceptualization, Investigation, Methodology, Software, Writing – original draft, Writing – review & editing. KY: Funding acquisition, Resources, Supervision, Validation, Visualization, Writing – review & editing. XC: Conceptualization, Data curation, Investigation, Methodology, Writing – review & editing. FX: Conceptualization, Formal analysis, Investigation, Methodology, Project administration, Writing – review & editing. FS: Methodology, Writing – review & editing, Conceptualization. XX: Data curation, Methodology, Writing – review & editing. TS: Data curation, Investigation, Methodology, Project administration, Writing – review & editing.
